# Comparison of effects between calcium channel blocker and diuretics in combination with angiotensin II receptor blocker on 24-h central blood pressure and vascular hemodynamic parameters in hypertensive patients: study design for a multicenter, double-blinded, active-controlled, phase 4, randomized trial

**DOI:** 10.1186/s40885-017-0074-0

**Published:** 2017-09-04

**Authors:** Gyu Chul Oh, Hae-Young Lee, Wook Jin Chung, Ho-Joong Youn, Eun-Joo Cho, Ki-Chul Sung, Shung Chull Chae, Byung-Su Yoo, Chang Gyu Park, Soon Jun Hong, Young Kwon Kim, Taek-Jong Hong, Dong-Ju Choi, Min Su Hyun, Jong Won Ha, Young Jo Kim, Youngkeun Ahn, Myeong Chan Cho, Soon-Gil Kim, Jinho Shin, Sungha Park, Il-Suk Sohn, Chong-Jin Kim

**Affiliations:** 1 Division of Cardiology, Department of Internal Medicine, Seoul National University Hospital, Seoul, Korea; 2 Division of Cardiovascular Disease, Department of Internal Medicine, Gachon University Gil Hospital, Medical Center, Incheon, Korea; 3 Department of Cardiology, Cardiovascular Center, Seoul St. Mary’s Hospital, Seoul, Korea; 4 Division of Cardiology, St. Paul’s Hospital, Seoul, Korea; 5 Division of Cardiology, Department of Medicine, Kangbuk Samsung Hospital, Seoul, Korea; 6 Division of Cardiology, Department of Internal Medicine, Kyungpook National University Hospital, Daegu, Korea; 7 Division of Cardiology, Department of Internal Medicine, Yonsei University Wonju Hospital, Wonju, Korea; 8 Department of Cardiology, Korea University Guro Hospital, Seoul, Korea; 9 Department of Cardiology, Cardiovascular Center, Korea University Anam Hospital, Seoul, Korea; 10 Division of Cardiology, Department of Internal Medicine, Dongguk University Ilsan Hospital, Goyang, Korea; 11 Division of Cardiology, Department of Internal Medicine, Pusan National University Hospital, Busan, Korea; 12 Cardiovascular Center, Division of Cardiology, Seoul National University Bundang Hospital, Seongnam, Korea; 13 Division of Cardiology, Department of Internal Medicine, Soonchunhyang Seoul Hospital, Seoul, Korea; 14 Division of Cardiology, Department of Internal Medicine, Severance Cardiovascular Hospital, Seoul, Korea; 15 Division of Cardiology, Department of Internal Medicine, Yeungnam University Hospital, Busan, Korea; 16 Division of Cardiology, Department of Internal Medicine, Chonnam National University Hospital, Gwangju, Korea; 17 Division of Cardiology, Department of Internal Medicine, Chungbuk National University Hospital, Cheongju, Korea; 18 Division of Cardiology, Department of Internal Medicine, Hanyang University Guri Hospital, Guri, Korea; 19 Division of Cardiology, Department of Internal Medicine, Kyung Hee University Hospital at Gangdong, Seoul, Korea; 20 Cardiovascular Center, Kyung Hee University Hospital at Gangdong, 892 Dongnam-ro, Gangdong-gu, Seoul, Korea

**Keywords:** Hypertension, Angiotensin II receptor blocker, Calcium channel blocker, Central blood pressure, Arterial stiffness, Fixed dose combination

## Abstract

**Background:**

Hypertension is a risk factor for coronary heart disease and stroke, and is one of the leading causes of death. Although over a billion people are affected worldwide, only half of them receive adequate treatment. Current guidelines on antihypertensive treatment recommend combination therapy for patients not responding to monotherapy, but as the number of pills increase, patient compliance tends to decrease. As a result, fixed-dose combination drugs with different antihypertensive agents have been developed and widely used in recent years. CCBs have been shown to be better at reducing central blood pressure and arterial stiffness than diuretics. Recent studies have reported that central blood pressure and arterial stiffness are associated with cardiovascular outcomes. This trial aims to compare the efficacy of combination of calcium channel blocker (CCB) or thiazide diuretic with an angiotensin receptor blocker (ARB).

**Methods:**

This is a multicenter, double-blinded, active-controlled, phase 4, randomized trial, comparing the antihypertensive effects of losartan/amlodipine and losartan/hydrochlorothiazide in patients unresponsive to treatment with losartan. The primary endpoint is changes in mean sitting systolic blood pressure (msSBP) after 4 weeks of treatment. Secondary endpoints are changes in msSBP, mean 24-h ambulatory mobile blood pressure, mean 24-h ambulatory mobile central SBP, mean 24-h ambulatory carotid-femoral pulse wave velocity, ambulatory augmentation index, and microalbuminuria/proteinuria after 20 weeks of treatment. The sample size will be 119 patients for each group in order to confer enough power to test for non-inferiority regarding the primary outcome.

**Conclusion:**

The investigators aim to prove that combination of a CCB with ARB shows non-inferiority in lowering blood pressure compared with a combination of thiazide diuretic and ARB. We also hope to distinguish the subset of patients that are more responsive to certain types of combination drugs. The results of this study should aid physicians in selecting appropriate combination regimens to treat hypertension in certain populations.

**Trial registration:**

ClinicalTrials.gov NCT02294539. Registered 12 November 2014.

## Background

Hypertension is known to be a major contributor to coronary heart disease and stroke, which ranks second and third in causes for death in Korea [1]. According to data from the Korea National Health and Nutrition Examination Survey (KNHANES), more than 50% of the Korean population over age 60 is prevalent with hypertension. For each 20/10 mmHg increase in systolic/diastolic blood pressure, the risk of coronary heart disease increases twofold. Treating high blood pressure prevents clinical events and saves lives [2, 3]. However, lowering blood pressure is not an easy task, and typically requires more than one drug to do the job.

Current guidelines on treating hypertension state that initial therapy should include a thiazide-type diuretic, calcium channel blocker (CCB), angiotensin-converting enzyme (ACE) inhibitor, or angiotensin receptor blocker (ARB) [4]. If the blood pressure goal is not met, doses should be increased or other classes should be added on to achieve the target blood pressure. More than 2/3 of patients with hypertension fail to achieve the goal with monotherapy, requiring multiple drugs with different mechanisms to achieve target blood pressure [5]. Combination therapy has been shown to enhance efficacy while reducing adverse events [6]. However, by increasing the number of drugs one has to take, there remains a risk of decreased compliance.

Fixed-dose combination (FDC) therapy for hypertension started in the 1960s with combination of hydrochlorothiazide (HCT) and triamterene, and since then different combinations using diuretics, CCBs, and renin-angiotensin system (RAS) inhibitors have been introduced [7, 8]. The rationale of FDC therapy is that lower doses of each drug can be used to reduce side effects while maintaining or even potentiating antihypertensive effects. In a study regarding Korean hypertensive patients, single pill therapy showed a significant increase in compliance and persistence, [9] which in turn leads to a decrease in cardiovascular morbidity and mortality.

RAS inhibitors combined with diuretics or CCBs are one of the recommended regimens for patients unresponsive to monotherapy. Combinations with either diuretics or CCBs provide synergistic blood pressure lowering effects and lower the probability of side effects. However, according to the ACCOMPLISH trial comparing the combination of ACE inhibitor with either CCB or HCT, the ACE inhibitor/CCB combo showed better cardiovascular outcomes in high-risk patients [10]. More recent trials on Japanese patients have also shown that ARB/CCB combination have similar effects on blood pressure and diastolic function compared with ARB/thiazide diuretic combinations [11, 12].

This study was designed to compare the antihypertensive effects of fixed-dose combination therapy of losartan/amlodipine (LST/AML) with that of losartan/hydrochlorothiazide (LST/HCT) in patients with hypertension unresponsive to monotherapy with losartan. In addition, this is the first study to compare the cardiovascular protective effects of two combinations, by monitoring 24-h blood pressure and pulse wave velocity, which have correlations with cardiovascular outcome.

## Methods/design

### Study objectives

The hypothesis of this trial is that fixed dose combination of losartan and amlodipine will be non-inferior to combination of losartan and hydrochlorothiazide in reducing blood pressure in patients with hypertension. Patient blood pressure was measured by conventional and ambulatory monitoring methods, and other vascular hemodynamic parameters were also obtained to test the hypothesis.

### Primary endpoint

The primary endpoint is the change in mean sitting systolic blood pressure (msSBP) after 4 weeks of treatment.

### Secondary endpoints

Secondary endpoints are changes in msSBP, mean 24-h ambulatory mobile blood pressure (AMBP), mean 24-h ambulatory mobile central SBP (AMcSBP), mean 24-h ambulatory carotid-femoral pulse wave velocity (AMPWV), ambulatory augmentation index (AMAIx), and microalbuminuria/proteinuria after 20 weeks of treatment. Treatment compliance, response rate, and success rate are also included in the secondary efficacy evaluation. Response to treatment is defined as a decrease in systolic blood pressure exceeding 20 mmHg or 10 mmHg for diastolic blood pressure, in accordance with the Korea Food and Drug Administration (KFDA). Treatment success is defined as blood pressure reaching a target of <140/90 mmHg.

### Blood pressure measurements

Blood pressure measurements will be obtained in the sitting position with the pressure cuff placed at either the right or left brachial area, using a semi-automated sphygmomanometer (HEM-7080IC, Omron Healthcare Co, Kyoto, Japan). After 5 min of rest, blood pressure will be measured 3 times with an interval of 2 min, and mean pressure will be used for analysis.

### Ambulatory monitoring and aortic stiffness measurement

Ambulatory measurements of peripheral and central blood pressure, carotid-femoral pulse wave velocity (cfPWV), and augmentation index (AIx) will be performed using a previously validated, automated oscillometric device (Mobil-O-graph 24 h PWA monitor, IEM Gmbh, Stolberg, Germany) [13–16].

### Study design

**Fig. 1:**
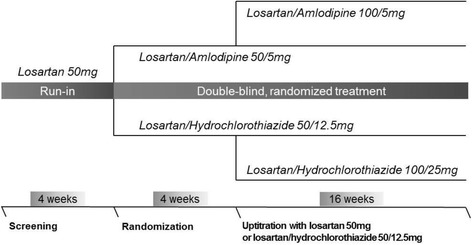
Study design

The trial has a multicenter, double-blinded, active-controlled, randomized design to compare the efficacy of fixed dose combination of losartan and amlodipine with that of losartan and hydrochlorothiazide. The detailed study design is shown in Fig. 1. Written consent will be obtained for all study patients, and patients will be permitted to request withdrawal from treatment at any time without providing reasons. The primary investigators and the attending physician will also have authority to drop out patients from the trial if it is considered that further participation would be detrimental to the patients’ well-being. After screening, patients eligible for the trial go through an open-labeled run-in period. Patients previously diagnosed with hypertension or those newly diagnosed are given losartan 50 mg daily for 4 weeks. The objective of the run-in period is to uncover patients not responding to a single class of antihypertensive treatment.

After 4 weeks of run-in, only patients with msSBP ≥140 mmHg will go through randomization and be given blinded treatment assignments. For the next 4 weeks, eligible patients are given either losartan/amlodipine 50/5 mg daily or losartan/hydrochlorothiazide 50/12.5 mg daily with a placebo drug of the other group. For patients not responding (msSBP ≥140 mmHg) to the first 4-week arm of the trial, doses will be increased to losartan/amlodipine 100/5 mg daily or losartan/hydrochlorothiazide 100/25 mg daily. The second arm of the trial will last for 16 more weeks. Patients responding to the initial dose will be maintained on their regimen.

Randomization is achieved by block randomization method to avoid bias and increase inter-group comparability. Block size is determined by an independent individual not participating in the trial. Patients are randomized 1:1 to either losartan/amlodipine or losartan/hydrochlorothiazide. Randomization results are kept in a sealed envelope and opened only in case of serious adverse events. Both the investigators and study participants are blinded to treatment group. To avoid treatments being visually distinguishable, the study drug and placebo are identical in appearance.

### Patient population

#### Inclusion criteria

Patients are to be recruited from 20 nationwide medical centers in Korea. Men and women from ages 19 to 80, with history of hypertension or those newly diagnosed with a systolic BP ≥140 mmHg are to be included in the study.

#### Exclusion criteria

The exclusion criteria are: (1) mean sitting diastolic blood pressure (msDBP) ≥110mgHg or msSBP ≥180 mmHg at screening or randomization; (2) variability of ≥20 mmHg in SBP or ≥10 mmHg in DBP between three measurements, or differences of ≥20/10 mmHg in left-to-right brachial values of SBP or DBP; (3) secondary hypertension; (4) malignant hypertension; (5) allergies or contraindications to ARB, CCB, or sulfonamides; (6) uncontrolled diabetes mellitus (HbA1c ≥10%); (7) history of New York Heart Association (NYHA) class III to IV heart failure, angina, myocardial infarction, cardiomyopathy, arrhythmia, or aortic stenosis requiring treatment within 6 months; (8) cerebral vascular disease within 6 months; (9) serious liver or renal dysfunction; (10) symptomatic hyperuricemia or gout; (11) galactose or lactose-intolerance; (12) patients with diabetes or moderate to severe renal dysfunction on drugs containing aliskiren; (13) pregnancy or the possibility of pregnancy, or breast feeding; (14) unable to withhold current medication; (15) prescription of other study drugs within 4 weeks; and (18) abnormal laboratory results (AST, ALT >3ULN, Cr >2.0 mg/dL, K+ <3.5 or >5.5 mEq/L, Na + <125 mEq/L, Protein >2+ on dipstick, or protein/creatinine >1000 mg/g on spot urine).

### Statistical analysis

#### Sample size

Sample size is calculated with 90% power to detect a difference in change of SBP of 3 mmHg between two groups at a two-sided significance level of 5%. To satisfy these assumptions and allowing for a drop-out rate of 20%, a total of 238 patients (119 for each treatment arm) are required for the trial.

#### Efficacy evaluation

Baseline characteristics will be compared using the Students t-test for continuous variables and the chi-square or Fisher’s exact test for categorical variables. Primary endpoint evaluation will be carried out using the two-sample t-test or Mann-Whitney test for inter-group comparison. For secondary endpoints, two-sample t-test and Wilcoxon’s rank sum test will be used for continuous variables, and the chi-square or Fisher’s exact test for categorical variables. Comparison of groups in achieving blood pressure target will be carried out using chi-square or McNemar’s test. All efficacy evaluations will be performed on the full analysis set (FAS). The FAS is a modified intention-to-treat set that includes patients receiving at least one dose of the study drug and having undergone at least one efficacy evaluation. Additional analysis will also be performed on the per protocol set. In order to minimize missing data, outcome data for patients who were discontinued from the study due to various reasons will be obtained and used for analysis. All investigators, sponsors, and regulators will aim to maximize the number of participants who are maintained on the assigned treatment until outcome data are collected.

#### Safety analysis

Safety analysis will be performed on all patients who has received the study drug at least once. Patients with self-reported or observed adverse symptoms will be seen by the attending physician and recorded according to treatment group and severity, and encoded to a system-organ class according to the Medical Dictionary for Regulatory Activities (MedDRA), version 16.0. The association with symptoms and drug treatment will also be evaluated. Abnormalities in laboratory results will be followed up until normalization and recorded for safety analysis.

## Results

Not applicable.

## Discussion

This study aims to compare the efficacy of two different types of losartan based combination drugs on blood pressure and vascular hemodynamic parameters. While previous studies have compared LOS/AML with LOS/HCT and have shown non-inferior antihypertensive results, [17] this trial will also investigate the effect two drugs have on central blood pressure and arterial stiffness. Central blood pressure and arterial stiffness are independent predictors of cardiovascular outcome, [18] and this is the first trial to study these parameters in losartan-based combination drugs.

The combination of ARB and CCB has proved to be beneficial beyond its additive BP reduction capabilities. ARBs selectively inhibit angiotensin II, which are potent vasoconstrictors. ARBs decrease blood pressure by vasodilation, and also decrease secretion of aldosterone, which in turn has protective effects in diabetic patients. CCBs relax smooth muscles and afferent arterioles in the kidney, leading to increased filtration in the glomerulus and finally resulting in reduced blood pressure. However, a well-known side effect of CCBs are peripheral edema and vasoconstriction. ARBs can reduce these side effects by antisympathetic effects and venous dilation. In addition, ARBs can lower the effect of increased renin-angiotensin-aldosterone (RAA) system, which rises from using CCBs [19]. It has also been reported that use of an ARB is associated with lower incidence of diabetes, while use of a diuretic is associated with a higher incidence [20]. The combination RAS inhibitor with a CCB is superior to a diuretic combination in avoiding the stimulation of the RAA system, reducing oxidative stress, reducing arterial stiffness, and slowing vascular aging [21]. In the ACCOMPLISH trial, combination with ACE inhibitor/CCB showed better outcomes compared to combination with ACE inhibitor/HCT [10]. ARBs may be preferred over ACE inhibitors as they have comparable antihypertensive effects and are associated with better tolerability.

The difference of this study compared to other non-inferiority trials is that we aim to measure noninvasive hemodynamic parameters in addition to conventional blood pressure. Conventional, clinic measured brachial blood pressure has its limitations. Clinic blood pressure does not reflect the daily peak-trough variations and can be masked by whitecoat hypertension. Furthermore, recent studies have shown that central blood pressure might be a better predictor of cardiovascular outcomes. According to the CAFE study, even with comparable reductions in brachial blood pressure, clinical outcome tends to differ due to the difference in reduction of central blood pressure [22]. While ACE inhibitors, ARBs, CCBs, and diuretics show comparable efficacy in lowering brachial blood pressure, diuretics are less effective than others in lowering central blood pressure because it lacks vasodilation abilities [23–25].

Arterial stiffness is also an useful tool to predict cardiovascular outcome and to evaluate subclinical organ damage in hypertensive patients [26]. Assessment of arterial stiffness can be accomplished by measuring the PWV or augmentation index from noninvasive monitoring of pulse waveforms [27]. Increased arterial stiffness results in increased systolic blood pressure and is independently associated with an increased risk of cardiovascular disease [28].

The investigators hypothesize that the blood pressure lowering effects of LOS/AML combination will be comparable to that of LOS/HCT while having fewer side effects. Additionally, through measuring central blood pressure, augmentation index, and PWV, this trial will test the hypothesis that while showing comparable blood pressure lowering effects, an ARB/CCB combination (losartan/amlodipine) has greater cardiovascular benefits than an ARB/diuretic combination (LOS/HCT).

## Conclusion

Current hypertension treatment guidelines strongly advocate the need to use combination therapy for patients not responsive to monotherapy. However, there is no clear directive on which combination would be most effective and also have beneficial cardiovascular outcomes. The importance of this study is that it will compare central blood pressure and vascular hemodynamic parameters that have correlation with clinical outcomes. The results of this trial should aid physicians in selecting the appropriate combination drug to treat hypertension in certain populations.

## References

[1] Korea, S (2015). Annual report on the causes of death statistics [internet].

[2] Five-year findings of the hypertension detection and follow-up program. I (1979). Reduction in mortality of persons with high blood pressure, including mild hypertension. Hypertension detection and follow-up program cooperative group. JAMA.

[3] Prevention of stroke by antihypertensive drug treatment in older persons with isolated systolic hypertension (1991). Final results of the systolic hypertension in the elderly program (SHEP). SHEP cooperative research group. JAMA.

[4] James PA (2014). 2014 evidence-based guideline for the management of high blood pressure in adults: report from the panel members appointed to the eighth joint National Committee (JNC 8). JAMA.

[5] Shin J (2015). 2013 Korean Society of Hypertension guidelines for the management of hypertension. Part II-treatments of hypertension. Clin Hypertens.

[6] Wald DS (2009). Combination therapy versus monotherapy in reducing blood pressure: meta-analysis on 11,000 participants from 42 trials. Am J Med.

[7] Epstein M, Bakris G (1996). Newer approaches to antihypertensive therapy. Use of fixed-dose combination therapy. Arch Intern Med.

[8] Weir MR, Bakris GL (2008). Combination therapy with Renin-Angiotensin-aldosterone receptor blockers for hypertension: how far have we come?. J Clin Hypertens (Greenwich).

[9] Min JY (2013). Compliance and persistence of free-combination antihypertensive therapy versus single-pill combination in Korean hypertensive patients. Int J Cardiol.

[10] Jamerson K (2008). Benazepril plus amlodipine or hydrochlorothiazide for hypertension in high-risk patients. N Engl J Med.

[11] Kondo K (2016). Comparison of telmisartan/amlodipine and telmisartan/hydrochlorothiazide in the treatment of Japanese patients with uncontrolled hypertension: the TAT-Kobe study. Blood Press Monit.

[12] Toh N (2016). Effect of diuretic or Calcium-Channel blocker plus Angiotensin-receptor blocker on diastolic function in hypertensive patients. Circ J.

[13] Wei W (2010). Validation of the mobil-O-graph: 24 h-blood pressure measurement device. Blood Press Monit.

[14] Weber T (2011). Validation of a brachial cuff-based method for estimating central systolic blood pressure. Hypertension.

[15] Hametner B (2013). Oscillometric estimation of aortic pulse wave velocity: comparison with intra-aortic catheter measurements. Blood Press Monit.

[16] Papaioannou TG (2013). Non-invasive 24 hour ambulatory monitoring of aortic wave reflection and arterial stiffness by a novel oscillometric device: the first feasibility and reproducibility study. Int J Cardiol.

[17] Suh SY (2014). Efficacy and tolerability of amlodipine camsylate/losartan 5/100-mg versus losartan/hydrochlorothiazide 100/12.5-mg fixed-dose combination in hypertensive patients nonresponsive to losartan 100-mg monotherapy. Clin Ther.

[18] Laurent S, Boutouyrie P (2007). Recent advances in arterial stiffness and wave reflection in human hypertension. Hypertension.

[19] Suarez C (2011). Single-pill telmisartan and amlodipine: a rational combination for the treatment of hypertension. Drugs.

[20] Elliott WJ, Meyer PM (2007). Incident diabetes in clinical trials of antihypertensive drugs: a network meta-analysis. Lancet.

[21] Ishimitsu T (2011). Antihypertensive therapy considering the prevention of vascular aging. J Korean Soc Hypertens.

[22] Williams B (2006). Differential impact of blood pressure-lowering drugs on central aortic pressure and clinical outcomes: principal results of the conduit artery function evaluation (CAFE) study. Circulation.

[23] Dahlof B (2005). Prevention of cardiovascular events with an antihypertensive regimen of amlodipine adding perindopril as required versus atenolol adding bendroflumethiazide as required, in the Anglo-Scandinavian cardiac outcomes trial-blood pressure lowering arm (ASCOT-BPLA): a multicentre randomised controlled trial. Lancet.

[24] Boutouyrie P (2010). Amlodipine-valsartan combination decreases central systolic blood pressure more effectively than the amlodipine-atenolol combination: the EXPLOR study. Hypertension.

[25] Jiang XJ (2007). Superior effect of an angiotensin-converting enzyme inhibitor over a diuretic for reducing aortic systolic pressure. J Hypertens.

[26] Taylor J (2013). 2013 ESH/ESC guidelines for the management of arterial hypertension. Eur Heart J.

[27] Rhee M-Y, Lee H-Y, Park JB (2008). Measurements of arterial stiffness: methodological aspects. Korean Circ J.

[28] Lee S-J, Park S-H (2013). Arterial Ageing. Korean Circ J.

